# Calculating pulmonary shunt fraction using standard clinical measurements

**DOI:** 10.14814/phy2.70763

**Published:** 2026-02-15

**Authors:** John G. Toffaletti, Gerald S. Zavorsky

**Affiliations:** ^1^ Department of Pathology Duke University Durham North Carolina USA; ^2^ Department of Physiology and Membrane Biology University of California at Davis Davis California USA

**Keywords:** blood gas analysis, hyperoxia, oxygen, oxygen consumption, pulmonary alveoli, respiratory function tests, shunt, physiologic, venous oxygen saturation

## Abstract

This single‐case pedagogical tutorial features a respiratory therapist who underwent a physiological shunt study for departmental education. Four formulas were used to estimate the shunt. A standardized 100% oxygen shunt test in a healthy male adult (51 years old, 185 cm, 84 kg) was performed to demonstrate how equation choice and assumptions affected the results. After 20 min breathing FiO_2_ = 1.0 at barometric pressure 752 mmHg, routine blood gas variables (PaO_2_ 587 mmHg, PaCO_2_ 38 mmHg, SaO_2_ 0.993, Hb 15.2 g/dL) were used to compute shunt by four approaches: the classic content equation, Chiang's arterial approximation, a P_A_O_2_–PaO_2_ rule‐of‐thumb, and a simplified saturation method. Across methods, estimates clustered between ~2.8% and 5.2%, illustrating close agreement in health yet revealing how dissolved oxygen, estimation of Sv̄O_2_, and assumed arterial–venous content shift results. The protocol and equations are presented as a teaching template for bedside calculation, with reporting of units, devices/supplies, and timing. We emphasize the disclosure of assumptions (RQ, nitrogen washout, capillary saturation), performing at least two methods when venous data are limited, and interpreting shunt results alongside clinical context. This practical, single‐case tutorial supports respiratory care/internal medicine education and strengthens confidence in the 100% oxygen shunt test.

## Graphical abstract

Physiologic shunt on 100% O_2_: convergent estimates in a healthy adult.

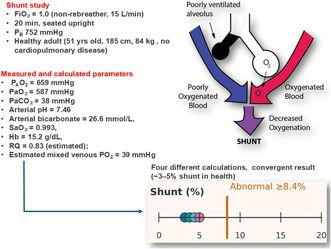

## 1 INTRODUCTION

Intrapulmonary shunt—the fraction of cardiac output that bypasses ventilated alveoli—remains a foundational concept in pulmonary physiology and clinical respiratory care. Because shunt cannot be measured directly, it is inferred from differences in oxygen content among end‐capillary, arterial, and mixed venous blood. Despite its importance, routine quantification is underused outside research and cardiothoracic anesthesia, largely because formulas are scattered across sources, units are inconsistently reported, and key assumptions are not always explicit. A widely used clinical strategy is to perform calculations after ~20 min of breathing 100% oxygen, when alveolar partial pressure of oxygen (P_A_O_2_) approximates end‐capillary PO_2_ and the dissolved oxygen terms can be ignored for simplification.

This short report reorganizes a familiar teaching scenario into a practical template for respiratory therapists and internal medicine physicians. We (1) demonstrate step‐by‐step shunt calculations in a healthy adult using only routine laboratory data; (2) compare four established approaches—classic content‐based calculation based on Riley's work from 1951 (Riley et al., 1951; Riley & Cournand, 1951); Chiang's (1968) simplified arterial approximation (Chiang, 1968); Ming's P_A_O_2_–P_a_O_2_ rule‐of‐thumb (Ming et al., 2014); and a saturation‐based approximation (Walley, 2011), and (3) highlight assumptions that govern accuracy and generalizability at the bedside. The goal is translational: to provide a concise, reproducible workflow that fits into routine practice and into teaching laboratories.

For clarity, we use “oxygenated” to describe blood oxygen content, and we reserve “saturation” for measured hemoglobin O_2_ saturation variables (e.g., S_a_O_2_, Sv̄O_2_).

## 2 METHODS

This is an educational clinical report that examines the various clinical formulas to calculate shunt by using a staff member's physiological results from a shunt study. One of the authors acted as the participant in the study and provided oral consent to publish. As an educational study, this was not considered research and did not need IRB approval. A controlled laboratory assessment was conducted at barometric pressure 752 mmHg. The subject was male, in his early fifties (185 cm, 84 kg) who had no cardiopulmonary disease. Baseline vital signs were stable (heart rate 55 beats/min; blood pressure 118/75 mmHg). The subject breathed 100% oxygen via a non‐rebreather mask at 15 L/min for 20 min while seated, with a respiratory therapist ensuring reservoir inflation. A right radial arterial sample was obtained using a 3 mL vented arterial sampling syringe with dry lithium heparin (Portex® Pro‐Vent). Air bubbles were expelled, and the sample was analyzed within 2 min of puncture with an ABL 90 Flex Plus blood‐gas analyzer (Radiometer, Copenhagen, DK). A modified Allen test (a bedside maneuver used to assess palmar arch patency/collateral ulnar circulation before radial artery puncture) was not performed because it has limited reliability for assessing collateral circulation (Pham et al., 2016). Puncture discomfort was modest and consistent with prior reports (Zavorsky et al., 2005).

Four approaches for calculating shunt are compared:

1. Equation 1—Classic shunt equation based on Riley's work (Riley et al., 1951; Riley & Cournand, 1951):

(1) $$\text{Shunt fraction}=\frac{{\dot{Q}}_{S}}{{\dot{Q}}_{T}}=\frac{C_{c\prime O_{2}}-C_{{\mathrm{aO}}_{2}}}{C_{c\prime O_{2}}-C_{\bar{v}O_{2}}}$$

The classic content equation defines the venous‐to‐arterial shunt fraction as *Q̇*_*s*_/*Q̇*_*T*_, where *Q̇*_*s*_ is shunted flow and *Q̇*_*T*_ is total cardiac output (*Q̇*_*T*_ = *Q̇*_*c*_ + *Q̇*_*s*_). If *Q̇*_*T*_ is measured, *Q̇*_*s*_ can be reported in absolute units; otherwise *Q̇*_*s*_/*Q̇*_*T*_ gives the proportion of cardiac output traversing the shunt (Chiang, 1968). The Cc'O_2_ is end‐capillary oxygen content, CaO_2_ is arterial oxygen content, and Cv̄O_2_ is mixed venous oxygen content.

$$\begin{array}{c} {\mathrm{CaO}}_{2}=1.34\;{\mathrm{mLO}}_{2}/g\times \left[\mathrm{Hb}\;\text{in}\;g/L\right]\times {\mathrm{SaO}}_{2}\\ +\left(0.003\;\mathrm{mL}\;O_{2}\;\mathrm{per}\;L\;\mathrm{per}\;\text{mmHg}\times {\mathrm{PaO}}_{2}\right), \end{array}$$

and

$$\begin{array}{c} C\bar{v}O_{2}=1.34\;{\mathrm{mLO}}_{2}/g\times \left[\mathrm{Hb}\;\text{in}\;g/L\right]\times S\bar{v}O_{2}\\ +\left(0.003\;\mathrm{mL}\;O_{2}\;\mathrm{per}\;L\;\mathrm{per}\;\text{mmHg}\times P\bar{v}O_{2}\right), \end{array}$$

After 20 min of breathing 100% oxygen, the alveolar partial pressure of oxygen (P_A_O_2_) should approximate the end‐capillary PO_2_ as there will be no gradient (Farhi & Rahn, 1955). Thus, Cc'O_2_ would be calculated as:

$${\mathrm{Cc}}^{'}O_{2}=\left(1.34\times \left[\mathrm{Hb}\right]\times 1.0\right)+\left(0.003\times P_{A}O_{2}\right)$$

When end‐capillary and peripheral blood are fully oxygenated (Hb O_2_ saturation ≈100%), the difference in their oxygen content is due to the oxygen in physical solution. Since the solubility of oxygen in whole blood is 0.0031 mL O_2_ / (100 mL blood · mmHg) PO_2_, then:

$$\begin{array}{c} \left({\mathrm{Cc}}^{'}O_{2}-{\mathrm{CaO}}_{2}\right)=\left(P_{A}O_{2}-P_{a}O_{2}\right)\times 0.003,\text{and}\;\left({\mathrm{Cc}}^{'}O_{2}-C\bar{v}O_{2}\right)\\ =\left(P_{A}O_{2}-P\bar{v}O_{2}\right)\times 0.003 \end{array}$$

2 Equation 2—Chiang (1968)–Simplified Arterial Equation:

This equation assumes a fixed arterial venous oxygen content difference of 5 mL/dL (Chiang, 1968).

This 5 mL/dL term represents a typical resting arteriovenous O_2_ content difference (i.e., whole‐body O_2_ extraction) in adults, which provides a convenient denominator when mixed venous sampling is unavailable. In critically ill patients, however, the arteriovenous O_2_ content difference can vary substantially with cardiac output, metabolic rate, and impaired extraction; therefore, Equation 2 should be interpreted as an approximation rather than a reference method.

Numerator:

$$\frac{\left(P_{A}O_{2}-P_{a}O_{2}\right)\times 0.003}{0.003}=P_{A}O_{2}-P_{a}O_{2}$$

Denominator:

$$\begin{array}{c} \frac{\left(P_{A}O_{2}-P_{a}O_{2}\right)\times 0.003+5}{0.003}=\left(P_{A}O_{2}-P_{a}O_{2}\right)\\ +\frac{5}{0.003}=\left(P_{A}O_{2}-P_{a}O_{2}\right)+1670 \end{array}$$

And then the simplified equation, Equation 2 is:

(2) $$\text{Shunt fraction}=\frac{{\dot{Q}}_{s}}{{\dot{Q}}_{t}}=\frac{P_{A}O_{2}-P_{a}O_{2}}{\left(P_{A}O_{2}-P_{a}O_{2}\right)+1670}$$

Equation 2 is a practical approximation of shunt fraction that can be calculated using just PO_2_ values, assuming 100% O_2_ breathing and fully oxygenated hemoglobin (Hb O_2_ saturation ≈100%). It avoids needing direct mixed venous samples or full content equations.

3 Equation 3—Ming et al. (2014) P_A_O_2_–PaO_2_ rule‐of‐thumb:

(3) $$\text{Shunt fraction}=\frac{P_{A}O_{2}-P_{a}O_{2}}{20}$$

After equilibrating on 100% O_2_, nitrogen washout minimizes *V̇*/*Q̇* mismatch, so the P_A_O_2_–PaO_2_ difference chiefly reflects shunt. Under normobaric hyperoxia, P_A_O_2_ ≈ 657–678 mmHg and, without anatomic shunt, PaO_2_ often exceeds 600 mmHg; any right‐to‐left flow mixes deoxygenated blood and lowers PaO_2_. Empirically, ~1% shunt decreases PaO_2_ by ~4 mmHg (~20 mmHg for 5%), yielding the bedside rule of Equation 3. This equation applies mainly on the flat portion of the oxyhemoglobin curve and only up to ~30% shunt; beyond that, nonlinearity leads to underestimation (Ming et al., 2014).

4 Equation 4—Simplified Saturation‐based Approximation (Walley, 2011):

(4) $$\text{Shunt fraction}=\frac{1-\mathrm{Sa}O_{2}}{1-S\bar{v}O_{2}}$$

Equation 4 is derived from Equation 1 but excludes the dissolved oxygen component. This simplification rests on several assumptions:

- Hemoglobin concentration [Hb] is constant across arterial, capillary, and venous blood, and oxygen content is assumed to be directly proportional to hemoglobin saturation.
- Dissolved O_2_ is negligible—a valid assumption under normal atmospheric FiO_2_, but not under 100% oxygen.
- End‐capillary oxygen content (Cc'O_2_) is approximated as fully oxygenated (Hb O_2_ saturation ≈ 100%).

Under these conditions, Equation 4 can provide a reliable estimate of the shunt (Walley, 2011). Using pulse oximetry for arterial oxygen saturation (SaO_2_), and central venous oxygen saturation (Sv̄O_2_)—assuming correct catheter placement and physiological stability (Walley, 2011)—shunt can be estimated with reasonable accuracy. This approach is beneficial when assessing tissue oxygenation, where Sv̄O_2_ offers more actionable insight than cardiac output (Walley, 2011). However, when evaluating cardiac function, echocardiography remains the superior tool (Walley, 2011).

### 2.1 Assumptions and constraints

**FIGURE 1:**
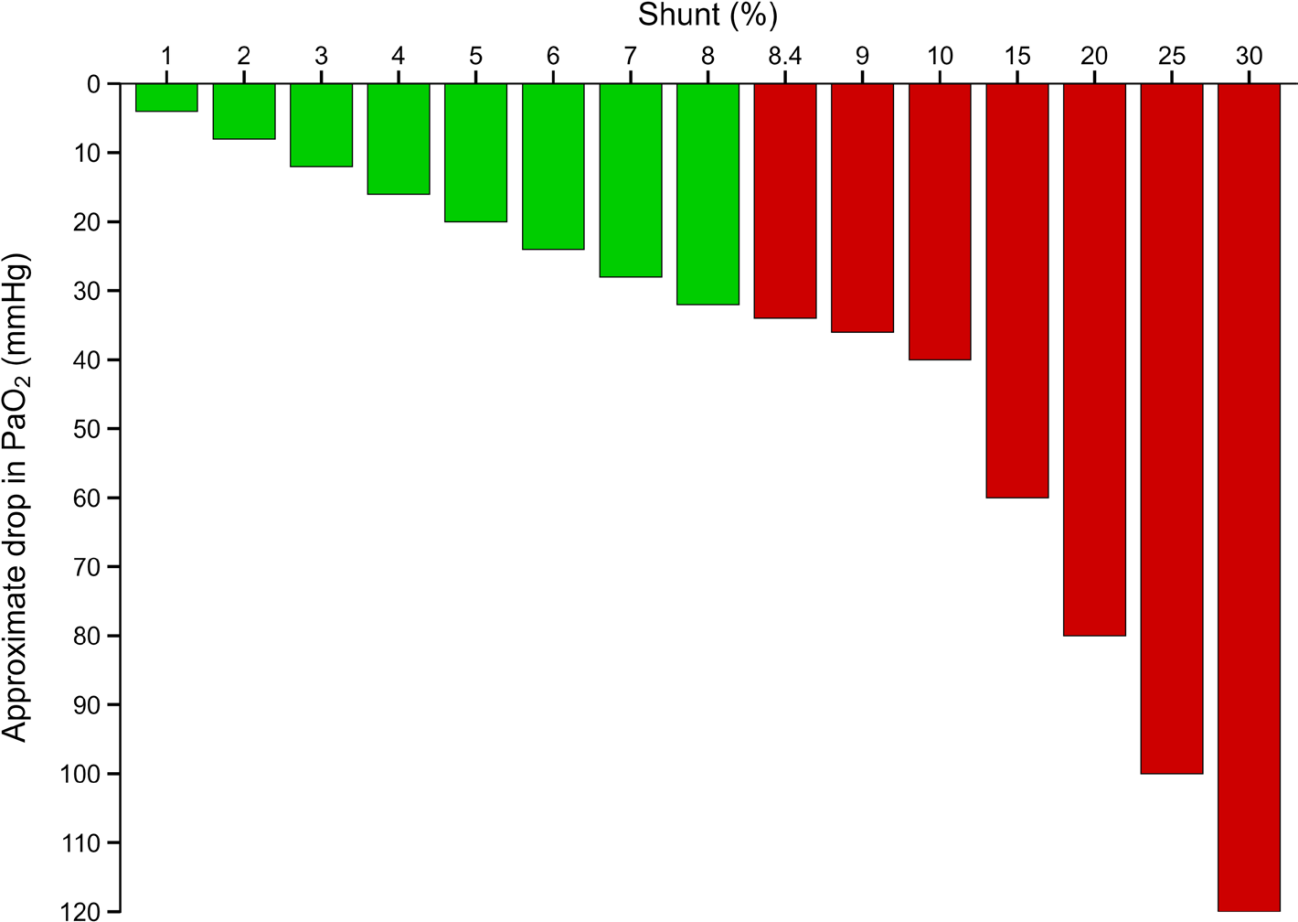
Estimated P_a_O_2_ reduction by shunt percentage during a standardized 100% O_2_ breathing test. Bars depict the expected P_a_O_2_ decrement from the alveolar partial pressure of oxygen (P_A_O_2_) as shunt fraction increases, using the bedside approximation from Ming et al. (2014) (≈4 mmHg P_a_O_2_ decrease per 1% shunt under normobaric hyperoxia, valid mainly up to ~30% shunt). Red bars highlight the proposed abnormal threshold of ≥8.4% (sensitivity 80%, specificity 75%; AUROC 0.70) from Ming et al. (2014). This graphic is intended as a teaching aid and should not be extrapolated to severe shunt where the P_a_O_2_–content relationship becomes nonlinear.

The 100% oxygen protocol assumes near‐complete pulmonary capillary saturation, stable Hb, and adequate nitrogen washout such that *V̇*/*Q̇* mismatch is minimized and the P_A_O_2_–P_a_O_2_ difference reflects shunt. Under these conditions, dissolved O_2_ terms are explicit and the relationship between PaO_2_ decrement and shunt is approximately linear up to ~30% shunt; beyond that range, nonlinearity of the O_2_ content–PaO_2_ curve degrades accuracy (Farhi & Rahn, 1955; Ming et al., 2014). A bedside lookup linking shunt percentage to the expected P_a_O_2_ decrement during 100% oxygen breathing is provided in Figure 1. This rule‐of‐thumb (~4 mmHg P_a_O_2_ decrement per 1% shunt) is most valid on the flat portion of the O_2_–Hb curve and should not be extrapolated beyond ~30% shunt. In external validation, an abnormal threshold of ≥8.4% yielded sensitivity of 80% and specificity of 75% (AUROC 0.70; *n* = 80) (Ming et al., 2014). Accordingly, Equations (2), (3), (4) are most reliable when shunt is low and calculations occur on the flat portion of the O_2_–Hb dissociation curve; as shunt increases, P_a_O_2_ may fall toward the steep portion of the curve and the assumed arteriovenous O_2_ content difference may change, leading to progressively larger bias (often underestimation) compared with the full content equation.

## 3 RESULTS

Measured inputs and derived parameters are summarized alongside formulas in Tables 1 and 2. The classic content equation yielded *Q̇*_*T*_/*Q̇*_*s*_ = 0.052 (5.2%). Across the four methods, shunt estimates ranged from approximately 2.8% (saturation‐based approximation (Walley, 2011)) to 5.2% (classic content equation based in Riley's work (Riley et al., 1951; Riley & Cournand, 1951)), indicating close agreement in this healthy subject.

**TABLE 1:** Measured and derived parameters after 20 min of 100% oxygen breathing in a male subject (51 years old, 185 cm tall, 84 kg).

Parameter	Value	Notes/Formula
Inspired oxygen fraction (FIO_2_)	1.00	
Barometric pressure (P_B_)	752 mmHg	
Respiratory quotient (RQ)	0.83 (estimated)	Mean from prior datasets (Goedecke et al., 2000; Zavorsky et al., 2005)
Arterial pH^a^	7.46	
Arterial bicarbonate (HCO_3_^−^)^a^	26.6 mmol/L	
Hemoglobin (Hb)	15.2 g/dL	
Arterial oxyhemoglobin saturation S_a_O_2_ (fraction)	0.993	
Arterial oxygen partial pressure (P_a_O_2_)	587 mmHg	
Arterial carbon dioxide partial pressure (P_a_CO_2_)	38 mmHg	
Estimated Pv̄O_2_ ^b^	39 mmHg	Peripheral venous PO_2_ + 5 mmHg; Sv̄O_2_ via standard equations (Severinghaus, 1966, 1979)
Sv̄O_2_ (estimated)^b^	0.746	

^a^ pH and HCO_3_^−^ results not needed in calculation of shunt fraction directly; however, they are used to estimate mixed venous oxygen saturation.

^b^ Mixed venous oxygen saturation (Sv̄O_2_) measured from the pulmonary artery provides the most comprehensive assessment of systemic oxygenation but requires invasive monitoring. Peripheral venous blood, such as from the antecubital vein, may be used to estimate Sv̄O_2_ using peripheral venous PO_2_. Add 5 mmHg to the peripheral venous PO_2_ to get an estimate of Pv̄O_2_. Then from this value, one can calculate Sv̄O_2_, using equations (Severinghaus, 1966, 1979). On the other hand, Sv̄O_2_, tends to be stable at rest, so estimates based on prior studies are often sufficient (Bevegard et al., 1960; Funaki et al., 2024). For example, across an estimated range of Sv̄O_2_ of 0.57–0.82, the calculated shunt fraction using the Simplified Saturation‐based Approximation (Equation 4) (Walley, 2011) would only vary from 1.6% (when Sv̄O_2_ =0.57) to 3.9% (when Sv̄O_2_ = 0.82) assuming all other variables remain constant.

**TABLE 2:** Calculated Parameters from data in Table 1 on an individual breathing 100% oxygen for 20 min. Subject is a male (51 years old, 185 cm tall, 84 kg).

Calculated parameter	Result
P_A_O_2_ = FIO_2_ × (pB − 47 mmHg) − (P_a_CO_2_ ÷RQ) = 1.0 × (752 − 47) − (38 ÷ 0.83)	659 mmHg
Arterial O_2_ physically dissolved in plasma 0.0031 mL O_2_ per L per mmHg × P_a_O_2_ = (0.0031 × 587)	1.82 mL/L
Mixed venous O_2_ physically dissolved in plasma 0.0031 mL O_2_ per L per mmHg × Pv̄O_2_ = (0.0031 × 39)	0.12 mL/L
Arterial O_2_ content (*C*aO_2_) C_a_O_2_ = 1.34 mLO_2_/g × [Hb in g/L] × S_a_O_2_ + (0.0031 mL O_2_ per L per mmHg × P_a_O_2_) = (1.34 × 15.2 × 0.993) + (0.0031 × 587)	22.05 mL/dL
Mixed venous O_2_ content (Cv̄O_2_) Cv̄O_2_ = 1.34 mLO_2_/g × [Hb in g/L] × Sv̄O_2_ + (0.0031 mL O_2_ per L per mmHg × Pv̄O_2_) = (1.34 × 15.2 × 0.746) + (0.0031 × 39)	15.31 mL/dL
End‐capillary O_2_ content (Cc'O_2_) Cc'O_2_ = 1.34 mLO_2_/g × [Hb in g/L] × S_a_O_2_ + (0.0031 mL O_2_ per L per mmHg × P_A_O_2_) = (1.34 × 15.2 × 1.00) + (0.0031 × 659)	22.41 mL/dL
O_2_ extraction (*C*aO_2_ − Cv̄O_2_) = 22.05 − 15.31	6.73 mL/dL
*Q̇*_*s*_ (shunted blood flow) = Cc'O_2_ − CaO_2_ = 22.41 − 22.05	0.360
*Q̇*_*t*_ (total blood flow) = Cc'O_2_ − Cv̄O_2_ = 22.41 − 15.31	7.10
*Q̇*_*s*_ */Q̇*_*t*_ = Shunted fraction = 0.360 ÷ 7.10	0.051 = 5.1%

*Note*: Mixed venous oxygen saturation (Sv̄O_2_): 0.746 (decimal fraction) either estimated from peripheral venous *p*O_2_ where the sO_2_ is from Severinghaus (1979) and the “x” is from Severinghaus (1966)
$${\mathrm{sO}}_{2}=100\cdot \frac{\left(x^{3}+150\cdot x\right)}{\left(x^{3}+150\cdot x+23,400\right)}\;\text{where}\;x={\mathrm{PO}}_{2}\cdot 10^{\left[0.48\cdot \left(\mathrm{pH}-7.40\right)-0.013\cdot \left({\mathrm{HCO}}_{3}-25\right)\right]}$$

The Bicarbonate (HCO_3_^−^): (26.6 mmol/L and pH = 7.46 is not used in shunt calculation but used in calculating x). Mixed venous PO_2_ = 39 mmHg. Note, In the online supplemental Excel file—Data S1, shunt fraction values are computed with full precision; accordingly, the value shown as 5.2% in the Excel file appears as 5.1% in this table due to rounding. These measurements were obtained using the protocol and blood‐gas analyzer described in Methods and were used to compute derived parameters (P_A_O_2_, C_a_O_2_, Cv̄O_2_, and Cc'O_2_) as shown in this table and Table 1.

## 4 DISCUSSION

**FIGURE 2:**
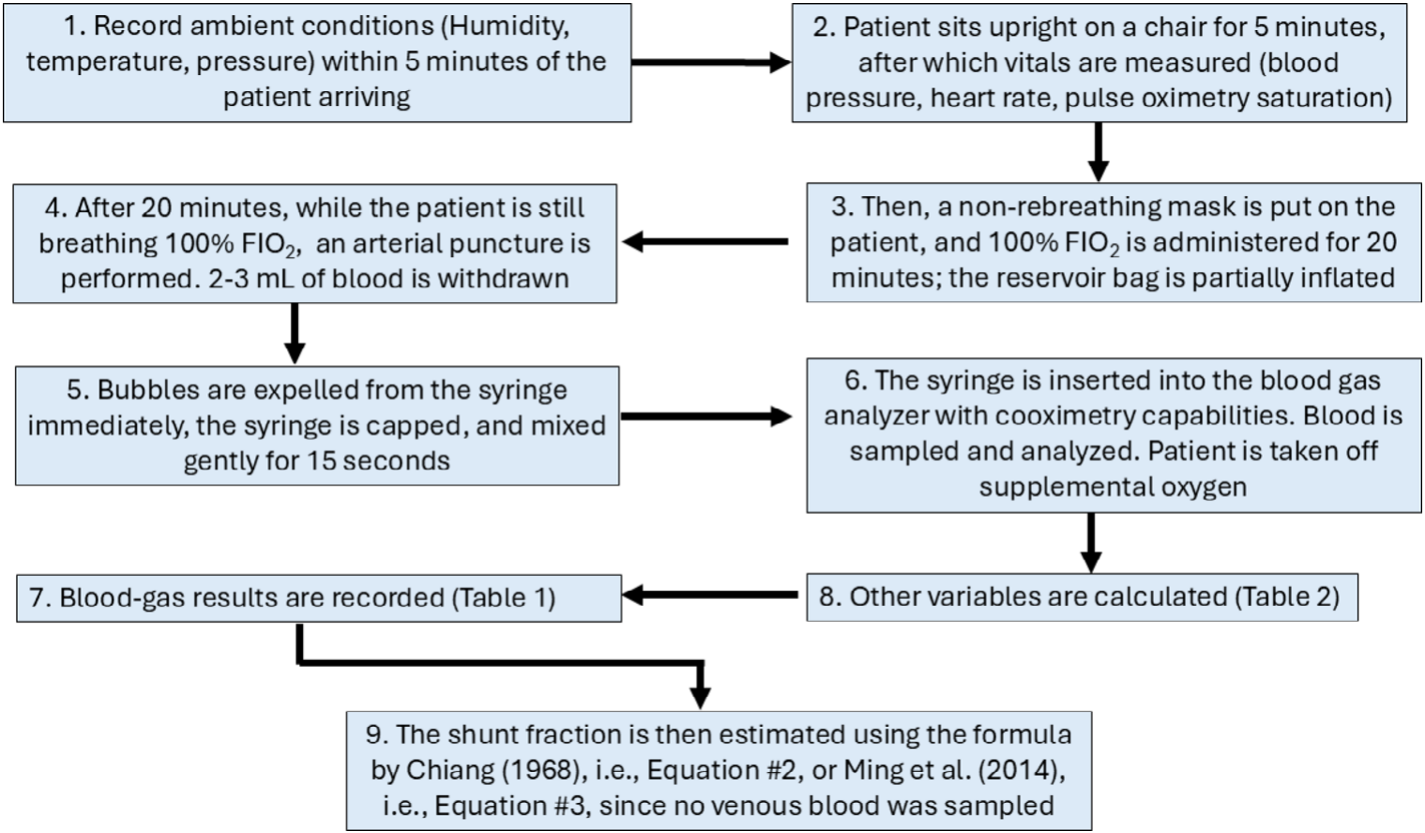
Standardized protocol for a 100% oxygen shunt study is used in this tutorial. After baseline assessment, the subject breathes FiO_2_ = 1.0 via non‐rebreather mask for 20 min with reservoir inflation ensured. An arterial sample is then obtained and analyzed promptly for P_a_O_2_, P_a_CO_2_, S_a_O_2_, and hemoglobin. P_A_O_2_ is calculated using the alveolar gas equation (with an assumed respiratory quotient), and shunt fraction is computed using the classic content equation and/or simplified approximations, with all assumptions explicitly stated.

This is a short pedagogical report that provides a compact, reproducible framework for estimating intrapulmonary shunt using routine measurements in a standardized hyperoxic protocol (Figure 2). The close agreement among four approaches (~2.8%–5.2%) in a healthy subject is consistent with physiologic shunt being predominantly anatomic—for example, bronchial and Thebesian venous return—under normobaric hyperoxia (Lenfant, 1964; Ravin et al., 1965). Clinically, the classic content equation is the reference method when mixed venous data are available or can be credibly estimated (Riley et al., 1951; Riley & Cournand, 1951); Chiang's arterial approximation and saturation‐based methods are useful when venous sampling is unavailable (Chiang, 1968), the P_A_O_2_–PaO_2_ rule‐of‐thumb provides rapid bedside triangulation with transparent assumptions (Ming et al., 2014), and the simplified saturation based approximation is beneficial when assessing tissue oxygenation, where Sv̄O_2_ offers more understanding than cardiac output (Walley, 2011). Accurate P_A_O_2_ depends on a reasonable RQ and on minimizing *V̇*/*Q̇* mismatch during 100% O_2_ administration. End‐capillary blood is assumed fully oxygenated (Hb O_2_ saturation ≈100%); Hb concentration is stable; and Sv̄O_2_ estimates based on peripheral venous PO_2_ should be contextualized (e.g., resting stability of Sv̄O_2_ and patient condition). Dissolved O_2_, though often small at room air, becomes non‐negligible in hyperoxia and is handled explicitly in the content equations.

### 4.1 Limitations

This tutorial uses a single healthy volunteer under controlled normobaric hyperoxia, so the close agreement among methods should not be assumed in patients with substantial shunt, unstable hemodynamics, or marked *V̇*/*Q̇* heterogeneity (e.g., ARDS, cardiogenic edema, intracardiac shunts). When shunt is suspected to be high or venous oxygenation is changing, the full content equation with directly measured Sv̄O_2_ remains the preferred reference, and simplified approaches (Equations (2), (3), (4)) should be used only as bedside triangulation with explicit assumptions.

Interpretation should integrate shunt with the broader clinical picture. As an orientation anchor rather than a rigid cutoff, ≥8.4% shunt on 100% O_2_ breathing has been proposed as abnormal (sensitivity 80%, specificity 75%) (Ming et al., 2014) (Figure 1). However, disease states such as acute respiratory distress syndrome (ARDS), cardiogenic edema, intracardiac shunts, or significant perfusion heterogeneity may violate underlying assumptions (e.g., incomplete nitrogen washout, reduced capillary transit time), which can bias estimates. In such contexts, pairing shunt calculations with imaging and hemodynamic assessments (e.g., echocardiography) is prudent, and central venous oximetry may guide therapy (Walley, 2011).

For respiratory therapists and clinicians, the practical value lies in standardized reporting: list measured variables with units; state formulas; document assumptions (e.g., RQ, device, timing to analysis); and present calculated content terms and *Q̇*_*T*_/*Q̇*_*s*_ transparently. Tables 1, 2 are designed as a didactic template that can be adapted for bedside calculations or incorporated into electronic worksheets (i.e., see online supplemental excel file). Future work should prospectively test simplified approximations across disease states and compare performance against content‐based calculations with directly measured Sv̄O_2_.

## 5 CONCLUSION

A standardized, 20‐min FiO_2_ = 1.0 protocol combined with routine blood gas variables allows respiratory therapists, clinicians and trainees to estimate intrapulmonary shunt with transparent assumptions. In this subject's case, four established methods yielded closely aligned values (~2.8%–5.2%). The classic content equation (Riley et al., 1951; Riley & Cournand, 1951) remains the reference when mixed venous data are available, whereas Chiang's approximation (Chiang, 1968), the P_A_O_2_–PaO_2_ rule‐of‐thumb (Ming et al., 2014), and a saturation‐based approach (Walley, 2011) provide practical alternatives when data are limited. Incorporating a simple reporting template and calculating shunt by at least two methods can improve robustness, aid bedside decision‐making, and enhance teaching in respiratory care and in internal medicine.

## AUTHOR CONTRIBUTIONS

Conceptualization (John G. Toffaletti, Gerald S. Zavorsky); methodology (John G. Toffaletti, Gerald S. Zavorsky); data collection and measurements (Gerald S. Zavorsky); formal analysis and calculations (John G. Toffaletti, Gerald S. Zavorsky); writing—original draft (John G. Toffaletti, GSZ); writing—review & editing (John G. Toffaletti, Gerald S. Zavorsky).

## FUNDING INFORMATION

The authors have nothing to report.

## CONFLICT OF INTEREST STATEMENT

The authors declare no conflicts of interest.

## ETHICS STATEMENT

As an educational study, this was not considered research and did not need IRB approval. One of the authors acted as the participant in the study and provided oral consent to publish.

## Supporting information


Data S1.

## Data Availability

All data is provided in this manuscript.
